# 2,2-Dimethyl-5-triphenyl­methyl-1,3-dioxane

**DOI:** 10.1107/S1600536809000294

**Published:** 2009-01-14

**Authors:** Min Zhang, Xian-You Yuan, Xing-Ming Liu

**Affiliations:** a Department of Biology and Chemistry, Hunan University of Science and Engineering, Yongzhou Hunan 425100, People’s Republic of China

## Abstract

The title compound, C_25_H_26_O_2_, crystallizes with two crystallographically independent mol­ecules in the asymmetric unit. The differences between the two mol­ecules are marginal. The three benzene rings of each mol­ecule are in a propeller orientation and the 1,3-dioxane ring adopts a chair conformation.

## Related literature

For the synthesis of the compound, see: Whilt & Finnerty (1961 ▶); Yuan *et al.* (2007 ▶); Wang *et al.* (1995 ▶). For applications of this class of compounds, see: Wang, Yuan, Liu *et al.* (1996 ▶); Wang, Yuan, Lei & Liu (1996 ▶); Yuan *et al.* (2005 ▶). For related crystal structures, see: Chuprunov *et al.* (1981 ▶); Yuan *et al.* (2008 ▶).
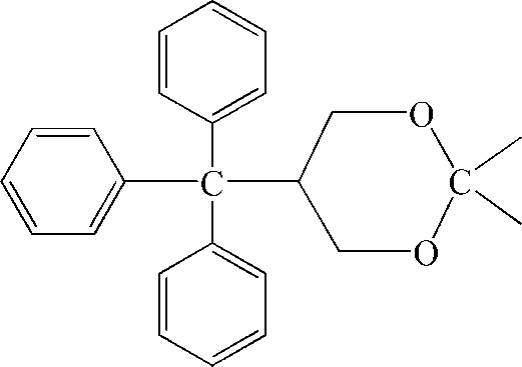


## Experimental

### 

#### Crystal data

C_25_H_26_O_2_
*M* *_r_* = 358.47
Triclinic, 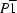

*a* = 10.7252 (18) Å
*b* = 11.6933 (19) Å
*c* = 15.840 (3) Å
α = 89.574 (3)°
β = 88.906 (3)°
γ = 86.427 (3)°
*V* = 1982.3 (6) Å^3^
*Z* = 4
Mo *K*α radiation
μ = 0.07 mm^−1^
*T* = 298 (2) K
0.45 × 0.38 × 0.29 mm

#### Data collection

Bruker SMART CCD area-detector diffractometer
Absorption correction: multi-scan (*SADABS*; Sheldrick, 1996 ▶) *T*_min_ = 0.967, *T*_max_ = 0.979
16710 measured reflections
8543 independent reflections
5142 reflections with *I* > 2σ(*I*)
*R*_int_ = 0.028

#### Refinement

*R*[*F* ^2^ > 2σ(*F* ^2^)] = 0.055
*wR*(*F* ^2^) = 0.193
*S* = 1.07
8543 reflections
491 parameters
H-atom parameters constrained
Δρ_max_ = 0.20 e Å^−3^
Δρ_min_ = −0.24 e Å^−3^

### 

Data collection: *SMART* (Bruker, 1997 ▶); cell refinement: *SAINT* (Bruker, 1997 ▶); data reduction: *SAINT*; program(s) used to solve structure: *SHELXS97* (Sheldrick, 2008 ▶); program(s) used to refine structure: *SHELXL97* (Sheldrick, 2008 ▶); molecular graphics: *SHELXTL* (Sheldrick, 2008 ▶); software used to prepare material for publication: *SHELXTL*.

## Supplementary Material

Crystal structure: contains datablocks I, global. DOI: 10.1107/S1600536809000294/bt2825sup1.cif

Structure factors: contains datablocks I. DOI: 10.1107/S1600536809000294/bt2825Isup2.hkl

Additional supplementary materials:  crystallographic information; 3D view; checkCIF report

## Figures and Tables

**Fig. 1.**
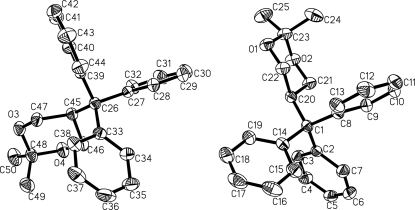
The molecular structure of the title compound, showing displacement ellipsoids at the 30% probability level. H-atoms have been excluded for clarity.

**Fig. 2.**
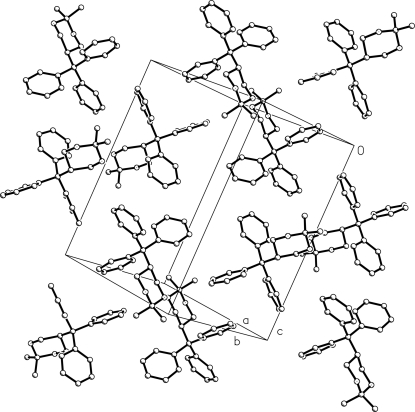
A view of the packing of the title compound.

## supplementary crystallographic information

### Comment

The title compound was synthesized to be use as a intermediate in surface activating reagent syntheses. The compound belongs to a class of 1,3-dioxane derivatives that have application in fine chemical medicine such as biology pharmacy (Wang, Yuan, Liu *et al.*, 1996) and cosmetic industry (Wang, Yuan, Lei & Liu, 1996; Yuan *et al.*, 2005).

The title compound  (Fig. 1) crystallizes with two crystallographically independent molecules per asymmetric unit. Differences between the two molecules are marginal. The three benzene rings of each molecule are in a propeller orientation and the 1,3-dioxane ring adopts a chair conformation. The structure is similar to that reported (Chuprunov *et al.*, 1981; Yuan *et al.*, 2008).

### Experimental

A mixture of 0.24 g (5.0 mmol) of 2,2-bis(hydroxymethyl)-1,3-propanediol, 0.10 g of phosphotungstic acid supported on actived carbon and 10 ml of acetone were added to a round bottom flask of 50 ml and heated under microwave irradiation condition for 3 min, then 5 ml benzene was added to this mixture. Then, this solution was heated, filtrated, and washed by using hot benzene. After benzene was evaporated and the sample was cooled. The resultant solid was recrystallized from anhydrous ethanol to give 1.1 g (58%) of white solid.

### Refinement

H atoms were placed in calculated positions (C—H 0.93 to 0.98 Å) and were included in the refinement in the riding model approximation, with *U*_iso_(H) set to 1.2  *U*_eq_(C) or 1.5 *U*_eq_(C_methyl_).

### Crystal data

**Table:**

C_25_H_26_O_2_	*Z* = 4
*M**_r_* = 358.47	*F*(000) = 768
Triclinic, *P*1	*D*_x_ = 1.201 Mg m^−^^3^
*a* = 10.7252 (18) Å	Mo *K*α radiation, λ = 0.71073 Å
*b* = 11.6933 (19) Å	Cell parameters from 5597 reflections
*c* = 15.840 (3) Å	θ = 2.2–27.0°
α = 89.574 (3)°	µ = 0.07 mm^−^^1^
β = 88.906 (3)°	*T* = 298 K
γ = 86.427 (3)°	Prism, colorless
*V* = 1982.3 (6) Å^3^	0.45 × 0.38 × 0.29 mm

### Data collection

**Table:**

Bruker SMART CCD area-detector diffractometer	8543 independent reflections
Radiation source: fine-focus sealed tube	5142 reflections with *I* > 2σ(*I*)
graphite	*R*_int_ = 0.028
φ and ω scans	θ_max_ = 27.2°, θ_min_ = 1.3°
Absorption correction: multi-scan (*SADABS*; Sheldrick, 1996)	*h* = −13→13
*T*_min_ = 0.967, *T*_max_ = 0.979	*k* = −14→14
16710 measured reflections	*l* = −20→20

### Refinement

**Table:**

Refinement on *F*^2^	Primary atom site location: structure-invariant direct methods
Least-squares matrix: full	Secondary atom site location: difference Fourier map
*R*[*F*^2^ > 2σ(*F*^2^)] = 0.055	Hydrogen site location: inferred from neighbouring sites
*wR*(*F*^2^) = 0.193	H-atom parameters constrained
*S* = 1.07	*w* = 1/[σ^2^(*F*_o_^2^) + (0.1105*P*)^2^] where *P* = (*F*_o_^2^ + 2*F*_c_^2^)/3
8543 reflections	(Δ/σ)_max_ < 0.001
491 parameters	Δρ_max_ = 0.20 e Å^−^^3^
0 restraints	Δρ_min_ = −0.24 e Å^−^^3^

### Special details

**Table:**

Geometry. All e.s.d.'s (except the e.s.d. in the dihedral angle between two l.s. planes) are estimated using the full covariance matrix. The cell e.s.d.'s are taken into account individually in the estimation of e.s.d.'s in distances, angles and torsion angles; correlations between e.s.d.'s in cell parameters are only used when they are defined by crystal symmetry. An approximate (isotropic) treatment of cell e.s.d.'s is used for estimating e.s.d.'s involving l.s. planes.
Refinement. Refinement of *F*^2^ against ALL reflections. The weighted *R*-factor *wR* and goodness of fit *S* are based on *F*^2^, conventional *R*-factors *R* are based on *F*, with *F* set to zero for negative *F*^2^. The threshold expression of *F*^2^ > σ(*F*^2^) is used only for calculating *R*-factors(gt) *etc*. and is not relevant to the choice of reflections for refinement. *R*-factors based on *F*^2^ are statistically about twice as large as those based on *F*, and *R*- factors based on ALL data will be even larger.

### Fractional atomic coordinates and isotropic or equivalent isotropic displacement parameters (Å^2^)

**Table:**

	*x*	*y*	*z*	*U*_iso_*/*U*_eq_	
O1	0.35990 (12)	0.60731 (12)	0.01269 (8)	0.0549 (4)	
O2	0.14384 (12)	0.63492 (12)	0.01747 (9)	0.0563 (4)	
C1	0.23664 (16)	0.38835 (15)	0.17018 (10)	0.0397 (4)	
C20	0.24344 (15)	0.49994 (15)	0.11561 (10)	0.0409 (4)	
H20	0.2502	0.5634	0.1550	0.049*	
C2	0.11404 (16)	0.39031 (15)	0.22318 (10)	0.0411 (4)	
C14	0.34210 (17)	0.37887 (16)	0.23626 (11)	0.0447 (4)	
C8	0.24895 (18)	0.28587 (15)	0.10870 (11)	0.0445 (4)	
C22	0.35745 (17)	0.49994 (17)	0.05630 (12)	0.0494 (5)	
H22A	0.4330	0.4868	0.0886	0.059*	
H22B	0.3543	0.4385	0.0158	0.059*	
C9	0.1479 (2)	0.25685 (17)	0.06129 (12)	0.0541 (5)	
H9	0.0701	0.2948	0.0705	0.065*	
C21	0.12987 (17)	0.53031 (17)	0.06175 (12)	0.0510 (5)	
H21A	0.1196	0.4694	0.0217	0.061*	
H21B	0.0555	0.5374	0.0976	0.061*	
C5	−0.1020 (2)	0.39266 (19)	0.32589 (12)	0.0564 (5)	
H5	−0.1744	0.3936	0.3592	0.068*	
C3	0.06172 (19)	0.49044 (17)	0.25835 (12)	0.0532 (5)	
H3	0.0995	0.5588	0.2481	0.064*	
C13	0.3626 (2)	0.22658 (18)	0.09322 (12)	0.0576 (5)	
H13	0.4319	0.2440	0.1240	0.069*	
C15	0.3606 (2)	0.27827 (19)	0.28297 (13)	0.0598 (5)	
H15	0.3095	0.2182	0.2740	0.072*	
C7	0.0561 (2)	0.29087 (17)	0.24326 (12)	0.0559 (5)	
H7	0.0897	0.2213	0.2223	0.067*	
C11	0.2745 (3)	0.1148 (2)	−0.01380 (14)	0.0774 (8)	
H11	0.2831	0.0581	−0.0547	0.093*	
C6	−0.0505 (2)	0.29260 (19)	0.29377 (13)	0.0628 (6)	
H6	−0.0876	0.2244	0.3059	0.075*	
C10	0.1616 (3)	0.1723 (2)	0.00074 (13)	0.0687 (7)	
H10	0.0930	0.1543	−0.0305	0.082*	
C4	−0.0455 (2)	0.49138 (19)	0.30843 (13)	0.0605 (6)	
H4	−0.0793	0.5603	0.3304	0.073*	
C12	0.3750 (3)	0.1417 (2)	0.03269 (14)	0.0727 (7)	
H12	0.4521	0.1025	0.0235	0.087*	
C16	0.4528 (2)	0.2656 (2)	0.34232 (14)	0.0753 (7)	
H16	0.4639	0.1970	0.3722	0.090*	
C19	0.4159 (2)	0.4675 (2)	0.25485 (13)	0.0666 (6)	
H19	0.4030	0.5374	0.2271	0.080*	
C18	0.5097 (3)	0.4537 (3)	0.31478 (16)	0.0946 (10)	
H18	0.5600	0.5138	0.3255	0.113*	
C17	0.5278 (2)	0.3528 (3)	0.35755 (15)	0.0896 (9)	
H17	0.5909	0.3434	0.3969	0.107*	
O3	0.83700 (13)	1.13075 (11)	0.47955 (8)	0.0561 (4)	
C26	0.77111 (16)	0.88371 (15)	0.33012 (11)	0.0409 (4)	
C39	0.88286 (16)	0.89755 (15)	0.26818 (11)	0.0414 (4)	
O4	0.62945 (13)	1.09080 (13)	0.49852 (9)	0.0633 (4)	
C27	0.65934 (17)	0.86683 (15)	0.27145 (11)	0.0426 (4)	
C45	0.74962 (16)	0.99479 (16)	0.38537 (11)	0.0442 (4)	
H45	0.7237	1.0581	0.3474	0.053*	
C40	0.88986 (19)	0.99737 (17)	0.22169 (12)	0.0516 (5)	
H40	0.8285	1.0562	0.2297	0.062*	
C33	0.79058 (18)	0.78071 (16)	0.39142 (11)	0.0466 (5)	
C47	0.86490 (18)	1.02937 (16)	0.43135 (12)	0.0493 (5)	
H47A	0.8942	0.9675	0.4686	0.059*	
H47B	0.9311	1.0427	0.3905	0.059*	
C46	0.64654 (18)	0.98545 (19)	0.45268 (13)	0.0571 (5)	
H46A	0.5690	0.9690	0.4260	0.069*	
H46B	0.6690	0.9231	0.4913	0.069*	
C29	0.5612 (2)	0.7445 (2)	0.17364 (13)	0.0642 (6)	
H29	0.5618	0.6753	0.1450	0.077*	
C28	0.6577 (2)	0.76446 (17)	0.22676 (12)	0.0535 (5)	
H28	0.7228	0.7089	0.2329	0.064*	
C38	0.9023 (2)	0.75861 (18)	0.43297 (12)	0.0561 (5)	
H38	0.9696	0.8023	0.4196	0.067*	
C44	0.9728 (2)	0.81094 (18)	0.25085 (13)	0.0578 (5)	
H44	0.9691	0.7411	0.2789	0.069*	
C41	0.9853 (2)	1.01248 (18)	0.16365 (13)	0.0593 (6)	
H41	0.9877	1.0810	0.1337	0.071*	
C43	1.0682 (2)	0.8258 (2)	0.19260 (14)	0.0672 (6)	
H43	1.1276	0.7660	0.1823	0.081*	
C42	1.0762 (2)	0.92683 (19)	0.15016 (13)	0.0608 (6)	
H42	1.1424	0.9375	0.1127	0.073*	
C32	0.5633 (2)	0.94808 (19)	0.25808 (12)	0.0587 (5)	
H32	0.5631	1.0182	0.2854	0.070*	
C34	0.6930 (2)	0.71336 (18)	0.41398 (12)	0.0578 (5)	
H34	0.6164	0.7263	0.3880	0.069*	
C31	0.4666 (2)	0.9269 (2)	0.20438 (14)	0.0721 (7)	
H31	0.4021	0.9827	0.1968	0.087*	
C30	0.4647 (2)	0.8250 (2)	0.16248 (14)	0.0706 (7)	
H30	0.3992	0.8108	0.1272	0.085*	
C36	0.8190 (3)	0.6071 (2)	0.51405 (15)	0.0844 (8)	
H36	0.8289	0.5491	0.5543	0.101*	
C37	0.9166 (3)	0.6740 (2)	0.49340 (14)	0.0753 (7)	
H37	0.9925	0.6618	0.5205	0.090*	
C35	0.7070 (3)	0.6273 (2)	0.47431 (14)	0.0750 (7)	
H35	0.6403	0.5830	0.4879	0.090*	
C48	0.73853 (19)	1.12060 (19)	0.54019 (13)	0.0584 (6)	
C23	0.25244 (18)	0.63539 (18)	−0.03605 (12)	0.0533 (5)	
C49	0.7751 (2)	1.0359 (2)	0.60968 (14)	0.0728 (7)	
H49A	0.7059	1.0295	0.6484	0.109*	
H49B	0.8451	1.0622	0.6393	0.109*	
H49C	0.7975	0.9623	0.5854	0.109*	
C25	0.2614 (2)	0.7575 (2)	−0.06423 (17)	0.0828 (8)	
H25A	0.2701	0.8051	−0.0158	0.124*	
H25B	0.1871	0.7825	−0.0937	0.124*	
H25C	0.3328	0.7630	−0.1012	0.124*	
C24	0.2427 (3)	0.5554 (2)	−0.11037 (13)	0.0809 (7)	
H24A	0.3168	0.5573	−0.1452	0.121*	
H24B	0.1712	0.5796	−0.1430	0.121*	
H24C	0.2339	0.4788	−0.0900	0.121*	
C50	0.7094 (2)	1.2394 (2)	0.57462 (16)	0.0812 (8)	
H50A	0.6878	1.2911	0.5291	0.122*	
H50B	0.7814	1.2647	0.6025	0.122*	
H50C	0.6405	1.2382	0.6142	0.122*	

### Atomic displacement parameters (Å^2^)

**Table:**

	*U*^11^	*U*^22^	*U*^33^	*U*^12^	*U*^13^	*U*^23^
O1	0.0381 (7)	0.0684 (9)	0.0581 (8)	−0.0039 (6)	−0.0018 (6)	0.0164 (7)
O2	0.0422 (7)	0.0641 (9)	0.0604 (8)	0.0094 (6)	0.0038 (6)	0.0162 (7)
C1	0.0353 (9)	0.0450 (10)	0.0387 (9)	−0.0001 (7)	−0.0037 (7)	−0.0040 (8)
C20	0.0343 (9)	0.0477 (10)	0.0406 (9)	−0.0013 (8)	−0.0018 (7)	−0.0016 (8)
C2	0.0416 (10)	0.0458 (10)	0.0359 (9)	−0.0038 (8)	−0.0026 (7)	−0.0009 (8)
C14	0.0411 (10)	0.0529 (11)	0.0393 (9)	0.0041 (8)	−0.0032 (8)	−0.0050 (8)
C8	0.0523 (11)	0.0433 (10)	0.0379 (9)	−0.0034 (8)	0.0014 (8)	−0.0046 (8)
C22	0.0365 (10)	0.0613 (12)	0.0495 (11)	0.0033 (9)	−0.0013 (8)	0.0096 (9)
C9	0.0586 (13)	0.0565 (12)	0.0487 (11)	−0.0149 (10)	−0.0026 (9)	−0.0047 (9)
C21	0.0358 (10)	0.0626 (12)	0.0543 (11)	−0.0014 (9)	−0.0027 (8)	0.0132 (10)
C5	0.0534 (12)	0.0716 (14)	0.0438 (11)	−0.0036 (10)	0.0080 (9)	0.0051 (10)
C3	0.0599 (13)	0.0456 (11)	0.0541 (11)	−0.0063 (9)	0.0131 (10)	−0.0037 (9)
C13	0.0608 (13)	0.0610 (13)	0.0500 (11)	0.0058 (10)	0.0011 (10)	−0.0096 (10)
C15	0.0651 (14)	0.0601 (13)	0.0528 (12)	0.0116 (10)	−0.0109 (10)	−0.0023 (10)
C7	0.0630 (13)	0.0496 (12)	0.0552 (12)	−0.0065 (10)	0.0076 (10)	−0.0024 (9)
C11	0.122 (2)	0.0614 (15)	0.0502 (13)	−0.0180 (15)	0.0145 (14)	−0.0183 (11)
C6	0.0662 (14)	0.0636 (14)	0.0600 (13)	−0.0201 (11)	0.0101 (11)	0.0029 (11)
C10	0.0908 (19)	0.0685 (15)	0.0502 (12)	−0.0308 (14)	−0.0022 (12)	−0.0122 (11)
C4	0.0671 (14)	0.0585 (13)	0.0541 (12)	0.0065 (11)	0.0136 (10)	−0.0037 (10)
C12	0.0911 (18)	0.0627 (14)	0.0613 (14)	0.0150 (13)	0.0144 (13)	−0.0119 (12)
C16	0.0769 (17)	0.0918 (18)	0.0531 (13)	0.0291 (15)	−0.0106 (12)	0.0094 (13)
C19	0.0656 (14)	0.0833 (16)	0.0538 (12)	−0.0241 (12)	−0.0184 (11)	0.0088 (11)
C18	0.0801 (18)	0.144 (3)	0.0663 (16)	−0.0493 (18)	−0.0329 (14)	0.0151 (17)
C17	0.0605 (16)	0.156 (3)	0.0525 (14)	−0.0076 (17)	−0.0195 (12)	0.0122 (17)
O3	0.0539 (8)	0.0572 (8)	0.0582 (8)	−0.0094 (6)	0.0013 (7)	−0.0184 (7)
C26	0.0394 (10)	0.0427 (10)	0.0406 (9)	−0.0018 (8)	−0.0012 (7)	−0.0035 (8)
C39	0.0398 (10)	0.0427 (10)	0.0420 (9)	−0.0046 (8)	−0.0009 (8)	−0.0058 (8)
O4	0.0431 (8)	0.0839 (10)	0.0627 (9)	0.0004 (7)	0.0009 (6)	−0.0299 (8)
C27	0.0412 (10)	0.0491 (10)	0.0383 (9)	−0.0082 (8)	−0.0007 (8)	0.0000 (8)
C45	0.0398 (10)	0.0494 (11)	0.0433 (10)	−0.0008 (8)	−0.0002 (8)	−0.0076 (8)
C40	0.0551 (12)	0.0476 (11)	0.0519 (11)	−0.0030 (9)	0.0043 (9)	−0.0021 (9)
C33	0.0514 (11)	0.0475 (11)	0.0405 (10)	−0.0005 (9)	0.0007 (8)	−0.0029 (8)
C47	0.0423 (10)	0.0527 (11)	0.0534 (11)	−0.0062 (8)	0.0038 (9)	−0.0145 (9)
C46	0.0407 (11)	0.0751 (14)	0.0565 (12)	−0.0095 (10)	0.0034 (9)	−0.0248 (10)
C29	0.0742 (16)	0.0693 (14)	0.0521 (12)	−0.0245 (12)	−0.0074 (11)	−0.0092 (11)
C28	0.0598 (13)	0.0521 (12)	0.0498 (11)	−0.0116 (10)	−0.0029 (9)	−0.0037 (9)
C38	0.0605 (13)	0.0566 (12)	0.0508 (11)	0.0013 (10)	−0.0087 (10)	−0.0056 (10)
C44	0.0615 (13)	0.0482 (11)	0.0626 (13)	0.0028 (10)	0.0121 (10)	0.0009 (10)
C41	0.0722 (15)	0.0529 (12)	0.0541 (12)	−0.0156 (11)	0.0066 (11)	0.0014 (10)
C43	0.0620 (14)	0.0647 (14)	0.0723 (15)	0.0099 (11)	0.0193 (12)	−0.0033 (12)
C42	0.0550 (13)	0.0701 (15)	0.0578 (12)	−0.0125 (11)	0.0150 (10)	−0.0062 (11)
C32	0.0546 (12)	0.0692 (14)	0.0518 (11)	0.0035 (11)	−0.0092 (10)	−0.0106 (10)
C34	0.0614 (13)	0.0649 (13)	0.0477 (11)	−0.0104 (11)	0.0058 (10)	0.0008 (10)
C31	0.0536 (13)	0.106 (2)	0.0555 (13)	0.0057 (13)	−0.0110 (10)	−0.0004 (13)
C30	0.0582 (14)	0.105 (2)	0.0509 (12)	−0.0175 (14)	−0.0097 (10)	−0.0061 (13)
C36	0.131 (3)	0.0755 (17)	0.0456 (13)	−0.0012 (17)	−0.0040 (15)	0.0143 (12)
C37	0.0946 (19)	0.0752 (16)	0.0547 (13)	0.0118 (14)	−0.0221 (13)	−0.0005 (12)
C35	0.099 (2)	0.0758 (16)	0.0509 (13)	−0.0183 (14)	0.0130 (13)	0.0066 (12)
C48	0.0465 (11)	0.0760 (15)	0.0531 (12)	−0.0049 (10)	0.0003 (9)	−0.0229 (11)
C23	0.0398 (11)	0.0672 (13)	0.0519 (11)	0.0037 (9)	0.0009 (9)	0.0150 (10)
C49	0.0682 (15)	0.0963 (18)	0.0542 (13)	−0.0077 (13)	0.0022 (11)	−0.0114 (13)
C25	0.0684 (16)	0.0839 (18)	0.0941 (18)	0.0031 (13)	0.0052 (14)	0.0387 (15)
C24	0.0796 (17)	0.114 (2)	0.0469 (12)	0.0107 (15)	−0.0044 (12)	0.0026 (13)
C50	0.0734 (17)	0.0898 (18)	0.0798 (16)	0.0063 (13)	−0.0030 (13)	−0.0411 (14)

### Geometric parameters (Å, °)

**Table:**

O1—C23	1.420 (2)	O4—C48	1.417 (2)
O1—C22	1.430 (2)	O4—C46	1.435 (2)
O2—C21	1.420 (2)	C27—C32	1.376 (3)
O2—C23	1.428 (2)	C27—C28	1.396 (3)
C1—C2	1.546 (2)	C45—C47	1.524 (2)
C1—C8	1.547 (2)	C45—C46	1.529 (3)
C1—C14	1.554 (2)	C45—H45	0.9800
C1—C20	1.565 (2)	C40—C41	1.381 (3)
C20—C21	1.523 (2)	C40—H40	0.9300
C20—C22	1.528 (2)	C33—C38	1.388 (3)
C20—H20	0.9800	C33—C34	1.388 (3)
C2—C3	1.383 (3)	C47—H47A	0.9700
C2—C7	1.384 (3)	C47—H47B	0.9700
C14—C19	1.379 (3)	C46—H46A	0.9700
C14—C15	1.391 (3)	C46—H46B	0.9700
C8—C13	1.383 (3)	C29—C30	1.368 (3)
C8—C9	1.391 (3)	C29—C28	1.378 (3)
C22—H22A	0.9700	C29—H29	0.9300
C22—H22B	0.9700	C28—H28	0.9300
C9—C10	1.382 (3)	C38—C37	1.375 (3)
C9—H9	0.9300	C38—H38	0.9300
C21—H21A	0.9700	C44—C43	1.383 (3)
C21—H21B	0.9700	C44—H44	0.9300
C5—C6	1.360 (3)	C41—C42	1.369 (3)
C5—C4	1.361 (3)	C41—H41	0.9300
C5—H5	0.9300	C43—C42	1.361 (3)
C3—C4	1.384 (3)	C43—H43	0.9300
C3—H3	0.9300	C42—H42	0.9300
C13—C12	1.384 (3)	C32—C31	1.389 (3)
C13—H13	0.9300	C32—H32	0.9300
C15—C16	1.378 (3)	C34—C35	1.386 (3)
C15—H15	0.9300	C34—H34	0.9300
C7—C6	1.383 (3)	C31—C30	1.369 (3)
C7—H7	0.9300	C31—H31	0.9300
C11—C10	1.364 (4)	C30—H30	0.9300
C11—C12	1.370 (4)	C36—C35	1.374 (4)
C11—H11	0.9300	C36—C37	1.377 (4)
C6—H6	0.9300	C36—H36	0.9300
C10—H10	0.9300	C37—H37	0.9300
C4—H4	0.9300	C35—H35	0.9300
C12—H12	0.9300	C48—C50	1.509 (3)
C16—C17	1.363 (4)	C48—C49	1.516 (3)
C16—H16	0.9300	C23—C25	1.501 (3)
C19—C18	1.398 (3)	C23—C24	1.518 (3)
C19—H19	0.9300	C49—H49A	0.9600
C18—C17	1.362 (4)	C49—H49B	0.9600
C18—H18	0.9300	C49—H49C	0.9600
C17—H17	0.9300	C25—H25A	0.9600
O3—C48	1.424 (2)	C25—H25B	0.9600
O3—C47	1.428 (2)	C25—H25C	0.9600
C26—C33	1.548 (3)	C24—H24A	0.9600
C26—C39	1.550 (2)	C24—H24B	0.9600
C26—C27	1.554 (2)	C24—H24C	0.9600
C26—C45	1.574 (2)	C50—H50A	0.9600
C39—C44	1.379 (3)	C50—H50B	0.9600
C39—C40	1.381 (2)	C50—H50C	0.9600
			
C23—O1—C22	114.18 (14)	C46—C45—H45	107.5
C21—O2—C23	114.44 (14)	C26—C45—H45	107.5
C2—C1—C8	112.14 (14)	C39—C40—C41	122.00 (19)
C2—C1—C14	104.69 (13)	C39—C40—H40	119.0
C8—C1—C14	110.69 (14)	C41—C40—H40	119.0
C2—C1—C20	111.03 (14)	C38—C33—C34	116.75 (18)
C8—C1—C20	107.10 (13)	C38—C33—C26	121.56 (17)
C14—C1—C20	111.27 (14)	C34—C33—C26	121.36 (18)
C21—C20—C22	106.52 (14)	O3—C47—C45	110.77 (15)
C21—C20—C1	115.43 (14)	O3—C47—H47A	109.5
C22—C20—C1	113.94 (14)	C45—C47—H47A	109.5
C21—C20—H20	106.8	O3—C47—H47B	109.5
C22—C20—H20	106.8	C45—C47—H47B	109.5
C1—C20—H20	106.8	H47A—C47—H47B	108.1
C3—C2—C7	116.45 (17)	O4—C46—C45	109.95 (16)
C3—C2—C1	121.60 (16)	O4—C46—H46A	109.7
C7—C2—C1	121.77 (16)	C45—C46—H46A	109.7
C19—C14—C15	117.11 (18)	O4—C46—H46B	109.7
C19—C14—C1	123.60 (18)	C45—C46—H46B	109.7
C15—C14—C1	119.21 (17)	H46A—C46—H46B	108.2
C13—C8—C9	117.53 (17)	C30—C29—C28	120.9 (2)
C13—C8—C1	121.48 (17)	C30—C29—H29	119.5
C9—C8—C1	120.75 (17)	C28—C29—H29	119.5
O1—C22—C20	110.11 (15)	C29—C28—C27	121.1 (2)
O1—C22—H22A	109.6	C29—C28—H28	119.5
C20—C22—H22A	109.6	C27—C28—H28	119.5
O1—C22—H22B	109.6	C37—C38—C33	121.9 (2)
C20—C22—H22B	109.6	C37—C38—H38	119.0
H22A—C22—H22B	108.2	C33—C38—H38	119.0
C10—C9—C8	120.9 (2)	C39—C44—C43	121.4 (2)
C10—C9—H9	119.6	C39—C44—H44	119.3
C8—C9—H9	119.6	C43—C44—H44	119.3
O2—C21—C20	110.71 (15)	C42—C41—C40	120.2 (2)
O2—C21—H21A	109.5	C42—C41—H41	119.9
C20—C21—H21A	109.5	C40—C41—H41	119.9
O2—C21—H21B	109.5	C42—C43—C44	120.9 (2)
C20—C21—H21B	109.5	C42—C43—H43	119.6
H21A—C21—H21B	108.1	C44—C43—H43	119.6
C6—C5—C4	118.78 (19)	C43—C42—C41	118.82 (19)
C6—C5—H5	120.6	C43—C42—H42	120.6
C4—C5—H5	120.6	C41—C42—H42	120.6
C2—C3—C4	121.57 (18)	C27—C32—C31	120.9 (2)
C2—C3—H3	119.2	C27—C32—H32	119.5
C4—C3—H3	119.2	C31—C32—H32	119.5
C8—C13—C12	121.1 (2)	C35—C34—C33	121.6 (2)
C8—C13—H13	119.5	C35—C34—H34	119.2
C12—C13—H13	119.5	C33—C34—H34	119.2
C16—C15—C14	121.5 (2)	C30—C31—C32	121.0 (2)
C16—C15—H15	119.3	C30—C31—H31	119.5
C14—C15—H15	119.3	C32—C31—H31	119.5
C6—C7—C2	121.51 (19)	C29—C30—C31	118.6 (2)
C6—C7—H7	119.2	C29—C30—H30	120.7
C2—C7—H7	119.2	C31—C30—H30	120.7
C10—C11—C12	119.2 (2)	C35—C36—C37	119.0 (2)
C10—C11—H11	120.4	C35—C36—H36	120.5
C12—C11—H11	120.4	C37—C36—H36	120.5
C5—C6—C7	120.88 (19)	C38—C37—C36	120.4 (2)
C5—C6—H6	119.6	C38—C37—H37	119.8
C7—C6—H6	119.6	C36—C37—H37	119.8
C11—C10—C9	120.8 (2)	C36—C35—C34	120.3 (2)
C11—C10—H10	119.6	C36—C35—H35	119.9
C9—C10—H10	119.6	C34—C35—H35	119.9
C5—C4—C3	120.77 (19)	O4—C48—O3	109.22 (15)
C5—C4—H4	119.6	O4—C48—C50	106.01 (17)
C3—C4—H4	119.6	O3—C48—C50	106.01 (18)
C11—C12—C13	120.6 (2)	O4—C48—C49	111.97 (19)
C11—C12—H12	119.7	O3—C48—C49	111.75 (18)
C13—C12—H12	119.7	C50—C48—C49	111.56 (18)
C17—C16—C15	120.5 (2)	O1—C23—O2	109.10 (15)
C17—C16—H16	119.8	O1—C23—C25	106.39 (17)
C15—C16—H16	119.8	O2—C23—C25	105.74 (17)
C14—C19—C18	121.0 (2)	O1—C23—C24	112.28 (17)
C14—C19—H19	119.5	O2—C23—C24	111.23 (18)
C18—C19—H19	119.5	C25—C23—C24	111.78 (19)
C17—C18—C19	120.2 (2)	C48—C49—H49A	109.5
C17—C18—H18	119.9	C48—C49—H49B	109.5
C19—C18—H18	119.9	H49A—C49—H49B	109.5
C18—C17—C16	119.6 (2)	C48—C49—H49C	109.5
C18—C17—H17	120.2	H49A—C49—H49C	109.5
C16—C17—H17	120.2	H49B—C49—H49C	109.5
C48—O3—C47	113.78 (14)	C23—C25—H25A	109.5
C33—C26—C39	113.51 (14)	C23—C25—H25B	109.5
C33—C26—C27	110.61 (14)	H25A—C25—H25B	109.5
C39—C26—C27	103.98 (13)	C23—C25—H25C	109.5
C33—C26—C45	107.38 (14)	H25A—C25—H25C	109.5
C39—C26—C45	109.49 (13)	H25B—C25—H25C	109.5
C27—C26—C45	111.94 (14)	C23—C24—H24A	109.5
C44—C39—C40	116.61 (17)	C23—C24—H24B	109.5
C44—C39—C26	123.42 (17)	H24A—C24—H24B	109.5
C40—C39—C26	119.74 (16)	C23—C24—H24C	109.5
C48—O4—C46	113.22 (15)	H24A—C24—H24C	109.5
C32—C27—C28	117.37 (18)	H24B—C24—H24C	109.5
C32—C27—C26	124.24 (16)	C48—C50—H50A	109.5
C28—C27—C26	118.35 (17)	C48—C50—H50B	109.5
C47—C45—C46	106.41 (14)	H50A—C50—H50B	109.5
C47—C45—C26	114.60 (14)	C48—C50—H50C	109.5
C46—C45—C26	113.10 (15)	H50A—C50—H50C	109.5
C47—C45—H45	107.5	H50B—C50—H50C	109.5
			
C2—C1—C20—C21	−51.71 (19)	C33—C26—C27—C32	130.26 (19)
C8—C1—C20—C21	71.02 (19)	C39—C26—C27—C32	−107.5 (2)
C14—C1—C20—C21	−167.89 (14)	C45—C26—C27—C32	10.6 (2)
C2—C1—C20—C22	−175.46 (14)	C33—C26—C27—C28	−52.2 (2)
C8—C1—C20—C22	−52.73 (19)	C39—C26—C27—C28	70.04 (19)
C14—C1—C20—C22	68.36 (18)	C45—C26—C27—C28	−171.86 (15)
C8—C1—C2—C3	−156.43 (17)	C33—C26—C45—C47	69.82 (19)
C14—C1—C2—C3	83.5 (2)	C39—C26—C45—C47	−53.8 (2)
C20—C1—C2—C3	−36.7 (2)	C27—C26—C45—C47	−168.60 (15)
C8—C1—C2—C7	28.6 (2)	C33—C26—C45—C46	−52.4 (2)
C14—C1—C2—C7	−91.5 (2)	C39—C26—C45—C46	−176.05 (16)
C20—C1—C2—C7	148.34 (17)	C27—C26—C45—C46	69.19 (19)
C2—C1—C14—C19	−108.4 (2)	C44—C39—C40—C41	−3.1 (3)
C8—C1—C14—C19	130.6 (2)	C26—C39—C40—C41	−177.85 (17)
C20—C1—C14—C19	11.6 (2)	C39—C26—C33—C38	45.1 (2)
C2—C1—C14—C15	68.2 (2)	C27—C26—C33—C38	161.54 (16)
C8—C1—C14—C15	−52.8 (2)	C45—C26—C33—C38	−76.1 (2)
C20—C1—C14—C15	−171.80 (16)	C39—C26—C33—C34	−141.63 (17)
C2—C1—C8—C13	−141.65 (18)	C27—C26—C33—C34	−25.2 (2)
C14—C1—C8—C13	−25.1 (2)	C45—C26—C33—C34	97.21 (19)
C20—C1—C8—C13	96.32 (19)	C48—O3—C47—C45	57.8 (2)
C2—C1—C8—C9	44.2 (2)	C46—C45—C47—O3	−54.4 (2)
C14—C1—C8—C9	160.70 (16)	C26—C45—C47—O3	179.86 (14)
C20—C1—C8—C9	−77.8 (2)	C48—O4—C46—C45	−59.9 (2)
C23—O1—C22—C20	−58.7 (2)	C47—C45—C46—O4	55.2 (2)
C21—C20—C22—O1	54.90 (19)	C26—C45—C46—O4	−178.10 (15)
C1—C20—C22—O1	−176.66 (14)	C30—C29—C28—C27	0.7 (3)
C13—C8—C9—C10	−0.4 (3)	C32—C27—C28—C29	−2.2 (3)
C1—C8—C9—C10	173.99 (17)	C26—C27—C28—C29	−179.92 (17)
C23—O2—C21—C20	57.8 (2)	C34—C33—C38—C37	0.2 (3)
C22—C20—C21—O2	−54.6 (2)	C26—C33—C38—C37	173.79 (18)
C1—C20—C21—O2	177.79 (14)	C40—C39—C44—C43	2.9 (3)
C7—C2—C3—C4	−2.3 (3)	C26—C39—C44—C43	177.44 (19)
C1—C2—C3—C4	−177.53 (18)	C39—C40—C41—C42	0.5 (3)
C9—C8—C13—C12	0.1 (3)	C39—C44—C43—C42	−0.2 (4)
C1—C8—C13—C12	−174.26 (18)	C44—C43—C42—C41	−2.5 (3)
C19—C14—C15—C16	−3.2 (3)	C40—C41—C42—C43	2.3 (3)
C1—C14—C15—C16	180.00 (18)	C28—C27—C32—C31	2.2 (3)
C3—C2—C7—C6	1.9 (3)	C26—C27—C32—C31	179.79 (18)
C1—C2—C7—C6	177.14 (18)	C38—C33—C34—C35	−0.8 (3)
C4—C5—C6—C7	−0.9 (3)	C26—C33—C34—C35	−174.35 (18)
C2—C7—C6—C5	−0.3 (3)	C27—C32—C31—C30	−0.7 (3)
C12—C11—C10—C9	0.2 (4)	C28—C29—C30—C31	0.8 (3)
C8—C9—C10—C11	0.3 (3)	C32—C31—C30—C29	−0.8 (4)
C6—C5—C4—C3	0.5 (3)	C33—C38—C37—C36	0.6 (3)
C2—C3—C4—C5	1.1 (3)	C35—C36—C37—C38	−0.9 (4)
C10—C11—C12—C13	−0.5 (4)	C37—C36—C35—C34	0.4 (4)
C8—C13—C12—C11	0.4 (3)	C33—C34—C35—C36	0.5 (3)
C14—C15—C16—C17	0.9 (3)	C46—O4—C48—O3	58.7 (2)
C15—C14—C19—C18	3.5 (3)	C46—O4—C48—C50	172.54 (18)
C1—C14—C19—C18	−179.8 (2)	C46—O4—C48—C49	−65.6 (2)
C14—C19—C18—C17	−1.7 (4)	C47—O3—C48—O4	−57.6 (2)
C19—C18—C17—C16	−0.8 (4)	C47—O3—C48—C50	−171.43 (17)
C15—C16—C17—C18	1.2 (4)	C47—O3—C48—C49	66.8 (2)
C33—C26—C39—C44	18.5 (2)	C22—O1—C23—O2	56.8 (2)
C27—C26—C39—C44	−101.8 (2)	C22—O1—C23—C25	170.43 (16)
C45—C26—C39—C44	138.43 (18)	C22—O1—C23—C24	−67.0 (2)
C33—C26—C39—C40	−167.18 (16)	C21—O2—C23—O1	−56.3 (2)
C27—C26—C39—C40	72.56 (19)	C21—O2—C23—C25	−170.34 (17)
C45—C26—C39—C40	−47.2 (2)	C21—O2—C23—C24	68.1 (2)
